# Functional magnetic neuroimaging data on age-related differences in task switching accuracy and reverse brain-behavior relationships

**DOI:** 10.1016/j.dib.2018.05.059

**Published:** 2018-05-18

**Authors:** Chandramallika Basak, Shuo Qin, Kaoru Nashiro, Margaret A. O’Connell

**Affiliations:** a School of Behavioral and Brain Sciences, University of Texas at Dallas, United States; b Center for Vital Longevity, University of Texas at Dallas, United States; c University of Southern California, United States

## Abstract

The data presented in this article is related to the research article entitled “Age-related Differences in BOLD Modulation to Cognitive Control Costs in a Multitasking Paradigm: Global Switch, Local Switch, and Compatibility-Switch Costs” (Nashiro et al., 2018) [1]. This article describes age-related differences in accuracies for various cognitive costs incurred during task switching across three different age-cohorts: younger (18–35 years), younger-old (50–64 years) and older-old (65–80 years). The cognitive costs evaluated were global switch costs (GSC), local switch costs (LSC) and compatibility switch costs (CSC). Whole brain analyses were conducted to determine the brain regions sensitive to these cognitive costs, irrespective of age. Furthermore, age-related differences in brain-behavior relationships were evaluated by correlating activations from these regions with global switch costs, indexed by both response times and accuracies, for younger and older adults separately. Activations of age-sensitive regions during the task, where younger adults activated more than the combined groups of older adults, were also correlated with response times and accuracies to determine age-related differences in brain-behavior relationships of these under-recruited brain regions by older adults.

**Specifications Table**

**Table:** 

*Subject area*	Psychology
*More specific subject area*	Cognitive Neuroscience
*Type of data*	Text file, Figures, Graphs
*How data was acquired*	Behavioral and imaging data were obtained using a Philips Achieva 3T MR scanner (Philips Medical Systems, Andover, MA, USA) with a 32-channel head coil.
*Data format*	Analyzed
*Experimental factors*	The between-subjects factor was either age-group (younger, older) or group (younger, younger-old, older-old), and the within-subjects factor was trial-type (Single, NS-comp, NS-incomp, SW-comp, SW-incomp) in ANOVAs. Except for Single, all other trial-types were obtained from Dual blocks. For the brain-behavior correlations, ROIs that were sensitive to two different type of cognitive costs, viz., Global Switch Cost (GSC) and Local Switch Cost (LSC), were first obtained, irrespective of age. The percent signal change from these ROIs were correlated with accuracies of their respective conditions for younger and older adults. That is, GSC ROIs were correlated with single and dual accuracies; LSC ROIs were correlated with non-switch and switch accuracies. Age-sensitive ROIs, indexed by younger > older contrast, for Task > Fixation were also determined, and then correlated with single and dual accuracies.
*Experimental features*	We used a hybrid blocked and event-related design. The task consisted of alternating cycles of task (T) and fixation (F) blocks with the following structure: F, T, F, T, F, T, F, T, F. Each fixation block was of 30 s duration and the four task blocks were of 154 s duration each. Each task block had 30 trials in which a stimulus was presented for 3 s, within which the participant responded, followed by a fixation cross. To optimize stimulus sequence and timing the inter-trial interval (ITI) ranged from 1.5 to 5 s with a mean ITI of 2.13 s. The first two task blocks were single task, the next two were dual task where the task for each trial was randomly selected from Odd/Even or High/Low tasks. Three type of cognitive control costs were evaluated in this study: global switch cost (GSC), local switch cost (LSC), and compatibility switch cost (CSC).
*Data source location*	Dallas, TX, USA
*Data accessibility*	Analyzed data is provided in this article.
*Related research article*	This data in brief article was submitted as a companion paper to a research article [1].

**Value of the data**

• The data presents accuracies of younger and older adults associated with performing single tasks and dual tasks, and age-related differences in brain activations for cost-sensitive regions.
• The associations between accuracy and recruitment of under-recruited brain regions by older adults for both single task and dual task were measured and could be compared with other ageing studies.
• Older adults compensatory brain recruitment patterns, associated with higher Dual accuracy, specifically in left middle frontal gyrus, left frontal pole, and cerebellar regions could be compared with other aging studies using different cognitive paradigms.

## 1 Data

The data analyses shared here include both behavioral and neuroimaging findings on age-related differences in cognitive control from a task switching paradigm [1]. The behavioral data is restricted to accuracy and the fMRI neuroimaging results are regarding brain activations. We also present relationships between accuracy and brain activations in younger and older adults to understand age-related differences in brain-behavior relationships.

## 2 Experimental design, materials, and methods

### 2.1 Imaging procedures

Scanning was performed with a Philips Achieva 3T MR scanner (Philips Medical Systems, Andover, MA, USA) with a 32-channel head coil. High-resolution anatomical images were acquired, using a transverse MPRAGE T1-weighted sequence with the following parameters (TR = 8.1 ms; TE = 3.7 ms; flip angle = 12°; acquisition matrix = 256 × 204; voxel size = 1 mm^3^; 160 slices). Functional images were acquired using an echo-planar sequence (TR = 2000 ms; TE = 30 ms; flip angle = 70°; acquisition matrix = 64 × 64; voxel size = 3.44 × 3.44 × 4 mm; 39 axial slices). We used a hybrid blocked and event-related design. The task consisted of alternating cycles of task (T) and fixation (F) blocks with the following structure: F, T, F, T, F, T, F, T, F. Each fixation block was of 30 s duration and the four task blocks were of 154 s duration each. Each task block had 30 trials in which a stimulus was presented for 3 s, within which the participant responded, followed by a fixation cross. To optimize stimulus sequence and timing the inter-trial interval (ITI) ranged from 1.5 to 5 s with a mean ITI of 2.13 s.

The first two task blocks were of single task, one requiring Odd/Even judgement of the shown digit against a pink background, another requiring a High/Low (> 5/< 5) judgment of the digit shown against a blue background. The next two blocks were of dual tasks, where the task for each trial was randomly selected from the Odd/Even task or the High/Low task. The digit 5 was never presented due to its ambiguity. Of the 60 dual task trials, 15 were non-switch compatible trials (NS-comp), 15 were switch compatible trials (SW-comp), 15 were non-switch incompatible trials (NS-incomp), and 15 were switch incompatible trials (SW-incomp). For non-switch (NS) trials, the task remained the same as the previous trial. For switch (SW) trials, the task was different from the previous trial. The stimulus-response mapping was consistent for the compatible (Comp) trials (i.e., same hand response was required for the stimulus shown, irrespective of the task requirements), but was inconsistent for the incompatible (Incomp) trials. This resulted in 30 trials each for NS, SW, Comp and Incomp conditions. GSC was assessed from Dual > Single contrast, LSC from the SW > NS contrast, and CSC from the Incomp > Comp contrast.

### 2.2 Imaging analyses: preprocessing

The first six echo-planar imaging volumes were not recorded to allow the signal to reach steady-state magnetization. Preprocessing were performed using FSL 5.0.4 (FMRIB׳s Software Library; www.fmrib.ox.ac.uk/fsl), which included motion correction with MCFLIRT [2], removal of non-brain structures using brain extraction technique (BET) [3], spatial smoothing of the data using a Gaussian kernel of 4 mm full-width at half-maximum, and high-pass temporal filtering equivalent to 380 s for block design analysis and 100 s for event-related design analysis. The lengths of high-pass filtering for the block and event-related designs were determined via the “estimate high-pass filter” function in FSL. We created a study-specific template by registering each participant׳s high-resolution structural image to 152 T1 Montreal Neurological Institute (MNI) using FLIRT (FMRIB׳s Linear Image Registration Tool), and subsequently smoothing and averaging these images into a composite image. We performed linear registration between the functional and structural images using affine boundary-based registration [4]. The structural images were then normalized to the study-specific template by linear registration (FLIRT tools from FSL, [2], [5]). The use of a hybrid design in this study enabled us to perform block and event-related analyses of the same data set, allowing for calculation of GSC related regions from the Single and the Dual blocks, and of LSC and CSC related regions from the trials within the Dual blocks.

### 2.3 Imaging analyses: cognitive cost-sensitive brain regions and its interactions with age

GSC: Whole-brain analyses were conducted using FSL FEAT 6.00. For each run in every participant, stimulus-dependent changes in BOLD signal were modeled with two regressors (i.e., single and dual task blocks). The fixation blocks were modeled as the baseline level of activity and therefore, were not included as a regressor. The regressors were convolved with a gamma hemodynamic response function, including the six head movement parameters as confounds. Temporal filtering was also applied. For the first-individual level analyses, the amplitude of the hemodynamic response was estimated to calculate GSC (dual blocks > single blocks). The resulting images were then entered into a group analysis to obtain the average activation for the Dual > Single contrast across all participants with ‘age’ as a covariate. *Z* (Gaussianised T/F) statistic images were thresholded at the whole-brain level using clusters determined by *z* > 2.33 and a corrected-cluster significance threshold of *p* = .01. This resulted in five clusters: 1) the left middle frontal gyrus, 2) the bilateral paracingulate cortex, 3) the right middle temporal gyrus, 4) the right frontal pole, and 5) left fronto-parietal cluster consisting of the left precentral gyrus and the left supramarginal gyrus (see Table 1).

**Table 1:** Brain regions associated with GSC and LSC based on whole brain analysis in the overall sample, after controlling for age effects.

				*MNI*					
*Cost*	*Contrast*	*H*	*Region*	*x*	*y*	*z*	*Z*	*Cluster Index*	*# of Voxels*
GSC	Dual > Single	L	Middle Frontal Gyrus	− 30	6	56	3.48	1	248
		L	Middle Frontal Gyrus	− 32	2	54	3.46	1	
		L	Precentral Gyrus	− 32	− 4	50	3.33	1	
		R	Paracingulate Gyrus	6	14	50	4.09	2	474
		L	Paracingulate Gyrus	− 4	18	48	3.63	2	
		R	Paracingulate Gyrus	10	22	46	3.48	2	
		R	Middle Temporal Gyrus	58	−48	− 10	3.54	3	607
		R	Middle Temporal Gyrus	42	− 32	− 6	3.43	3	
		R	Inferior Temporal Gyrus	54	− 34	−12	3.36	3	
		R	Middle Frontal Gyrus	46	22	36	3.97	4	1494
		R	Frontal Pole	32	44	2	3.83	4	
		R	Frontal Pole	38	40	34	3.79	4	
		L	Precentral Gyrus	− 42	6	32	5.88	5	14,784
		L	Supramarginal Gyrus	− 48	− 46	44	5.52	5	
		L	Lateral Occipital Cortex	− 32	− 68	46	5.21	5	
									
LSC	SW > NS	R	Postcentral Gyrus	40	− 28	54	3.39	1	241
		R	Postcentral Gyrus	48	− 18	50	3.14	1	
		R	Precentral Gyrus	36	− 22	52	3.09	1	

LSC and CSC: For each run in every participant, stimulus-dependent changes in BOLD signal were modeled with six regressors: 1) Single, 2) NS-comp, 3) NS-incomp, 4) SW-comp, 5) SW-incomp, and 6) error trials. The regressors were convolved with a gamma hemodynamic response function, including the six head movement parameters as confounds. Temporal filtering was applied, and temporal derivatives of each of the regressors were also included. LSC regions were determined by the difference between switch trials (SW) and non-switch trials (NS), and were represented by the SW > NS contrast. CSC regions were determined by the difference between incompatible and compatible trials, and were represented by the Incomp > Comp contrast. The resulting images were then entered into the group analyses to obtain an average across all participants; ‘age’ was included as a covariate. *Z* (Gaussianised T/F) statistic images were thresholded at the whole-brain level using clusters determined by *z* > 2.33 and a corrected-cluster significance threshold of *p* = .01. Because no significant clusters were observed for either LSC or CSC at *p* < .01, the threshold for corrected-cluster significance was lowered to *p* = .05. Although no regions were identified for CSC, a cluster (right precentral and postcentral gyri) was identified for LSC region (see Table 1).

**Fig. 1:**
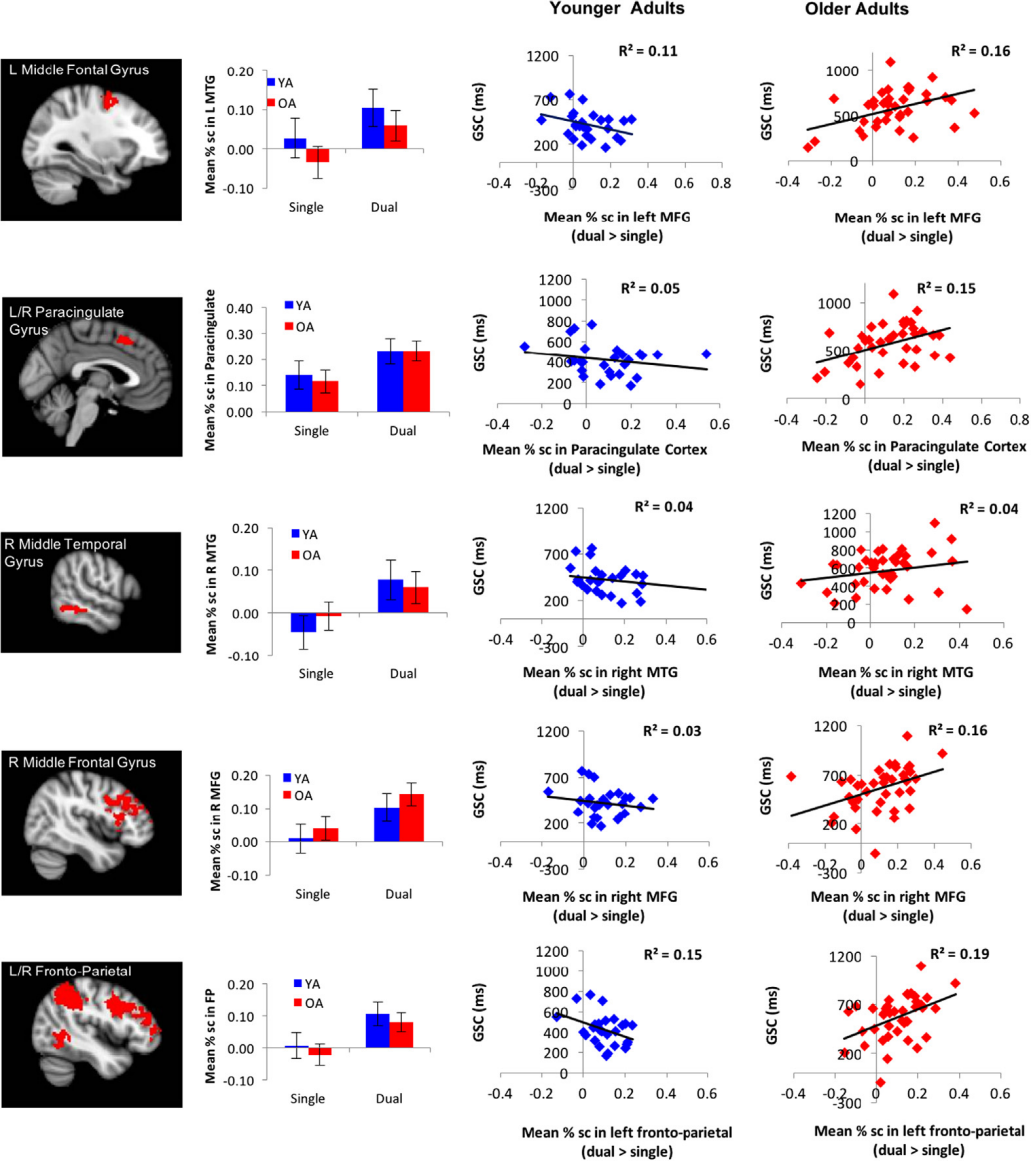
Age-group × condition (Dual vs. Single) interactions were non-significant in all five GSC-sensitive brain regions: left middle frontal gyrus, bilateral paracingulate gyrus, right middle temporal gyrus, right middle frontal gyrus, and bilateral fronto-parietal cluster.

For each of the 5 GSC regions, a 2 (age-group: younger vs. older) x 2 (condition: single vs. dual) ANOVA was conducted. As expected, we found a main effect of condition for all five GSC-sensitive regions, suggesting greater activation in dual than single blocks: the left middle frontal gyrus (*F*(1, 65) = 22.27, *MSE* = .01, *p* < .001, *η*_*p*_^*2*^ = .26), the bilateral paracingulate (*F*(1, 65) = 26.84, *MSE* = .01, *p* < .001, *η*_*p*_^*2*^ = .29), the right middle temporal gyrus (*F*(1, 65) = 25.51, *MSE* = .01, *p* < .001, *η*_*p*_^*2*^ = .28), the right middle frontal gyrus (*F*(1, 65) = 32.24, *MSE* = .01, *p* < .001, *η*_*p*_^*2*^ = .33), and the left fronto-parietal regions (*F*(1, 65) = 60.36, *MSE* = .01, *p* < .001, *η*_*p*_^*2*^ = .48). However, there were no significant main effects of age-group (*p* > .39) or age-group × condition interactions (*p* > .14) in any of the five GSC-sensitive regions. Fig. 1 depicts the data from all of these five regions.

### 2.4 Imaging analyses: age-sensitive brain regions favoring younger adults

**Fig. 2:**
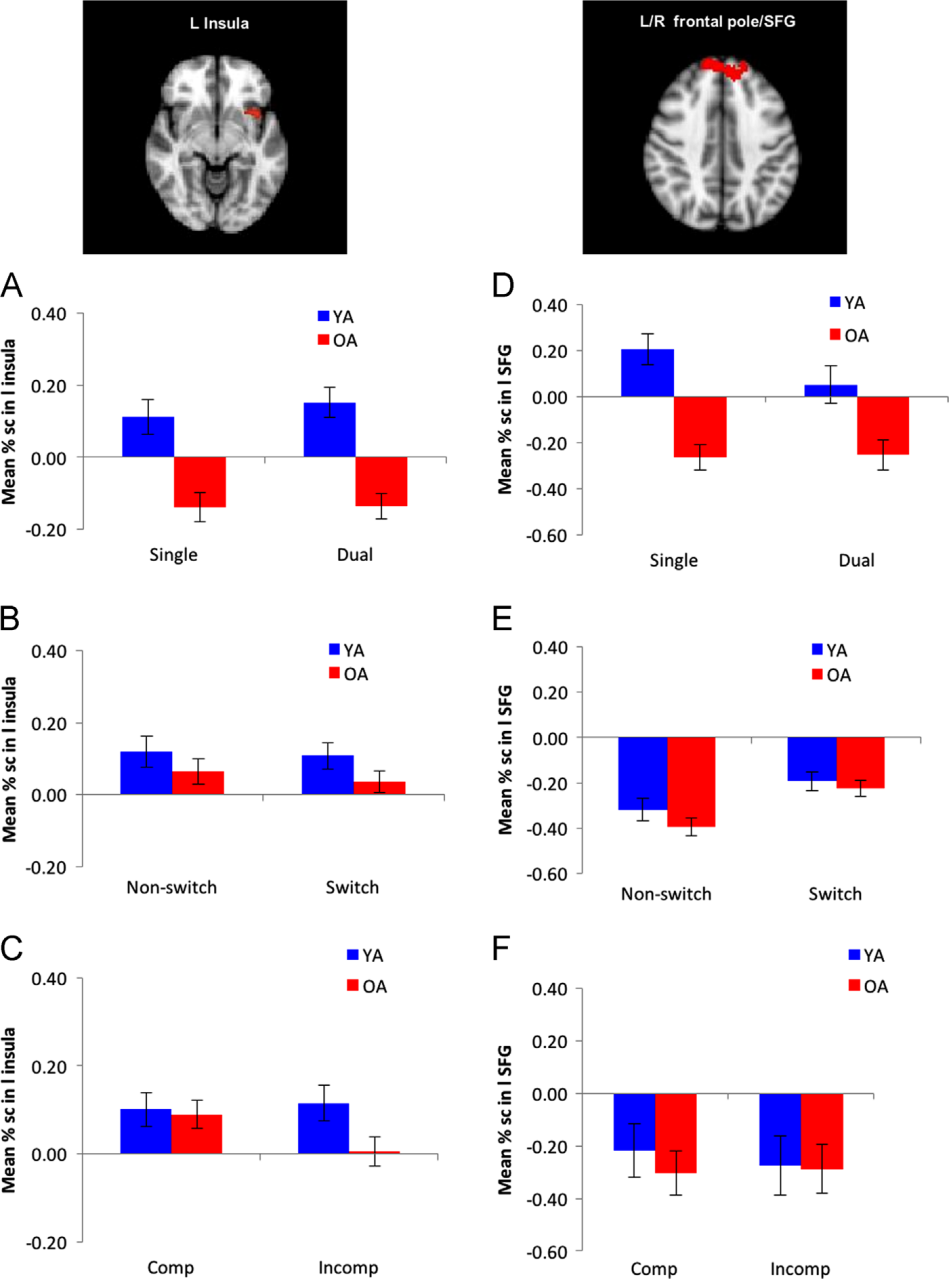
Age-group differences in age-sensitive regions (younger > older contrast) for different types of cognitive control mechanisms. A) Left Insula: non signifiicant age-group × condition (Dual > Single) interaction. B) Left Insula: non significant age-group x condition (NS vs. SW) interaction were significant for the left insula. C) Left:Insula: significant age-group and condition (Incomp vs. Comp) interaction. D) Bilateral frontal pole/SFG: significant age-group × condition (dual vs. single) interaction. E) Bilateral frontal pole/SFG: non significant age-group × condition (NS vs. SW) interaction. F) Bilateral frontal pole/SFG: non significant age-group × condition (Incomp vs. comp) interaction.

Group analyses were conducted to determine the brain regions that showed age-related differences in under recruitment by older adults (YA > OA) for Task > Fixation contrast, where clusters were determined at whole-brain level by *z* >2.33. A corrected-cluster significance threshold of *p* = .01 was used. Two clusters were identified for younger adults’ over-recruitment during the task compared to the older adults: the left insula and the bilateral frontal pole/SFG (see Table 1). Fig. 2 depicts the percent signal change for the three types of cognitive control (global switch costs, local switch costs, and compatibility costs) for both left insula (A, B and C) and bilateral frontal pole/SFG (D, E, and F). The statistical results associated with these figures are reported in the main manuscript [1].

### 2.5 Imaging analyses: whole brain regression analyses in older adults with dual accuracy as the predictor

In order to determine brain regions associated with better performance in older adults, we conducted three separate whole brain regression analyses for each cost-sensitive contrast (Dual > Single, SW > NS, Incomp > Comp), with Dual accuracy as the predictor. Seven clusters from the Dual > Single contrast were identified as being positively predicted by Dual accuracy in older adults (Table 2). For each of the 7 regions, a 2 (age-group: young vs. old) × 2 (condition: Single vs. Dual) ANOVA was conducted with no significant age-group effects (see *F* statistics in Table 2). No significant regions for SW > NS and Incomp > Comp contrasts were observed.

**Table 2:** GSC-sensitive brain regions (from Dual > Single contrast) associated with Dual accuracy in older adults, resulting from whole brain correlation analysis.

		*MNI*						
*H*	*Region*	*x*	*y*	*z*	*Z*	*Cluster Index*	*# of Voxels*	*F (1,65)*
L	Precentral Gyrus	− 42	8	30	3.61	1	254	17.8*
L	Middle Frontal Gyrus	− 48	10	34	3.24	1		
L	Inferior Frontal Gyrus	− 54	12	26	2.98	1		
L	Frontal Pole	− 40	44	18	3.65	2	281	37.13*
L	Middle Frontal Gyrus	− 46	22	30	3.44	2		
L	Inferior Frontal Gyrus	− 26	34	10	3.21	2		
L	Precuneous	− 6	− 70	46	3.54	3	467	19.69*
L	Precuneous	0	− 72	46	3.44	3		
R	Precuneous	18	− 74	48	3.17	3		
R	Cerebellum	2	− 76	− 28	3.56	4	469	11.46
R	Cerebellum	2	− 80	− 28	3.52	4		
R	Cerebellum	4	− 74	− 40	3.31	4		
R	Cerebellum	34	− 74	− 20	3.95	5	674	4.73
R	Cerebellum	28	− 70	− 30	3.8	5		
R	Cerebellum	36	− 56	− 24	3.67	5		
R	Middle Frontal Gyrus	46	24	36	4.08	6	874	7.9
R	Inferior Frontal Gyrus	52	16	28	3.45	6		
R	Middle Frontal Gyrus	46	28	26	3.42	6		
L	Lateral Occipital Cortex	− 36	− 68	44	3.89	7	1116	67.72*
L	Superior Parietal Lobule	− 30	− 52	42	3.83	7		
L	Lateral Occipital Cortex	− 30	− 62	46	3.65	7		

Note. *F* denote the condition main effect *F* statistic from repeated measures ANOVA. All *F* statistics were significant at *p* < .05, * denotes *p* < .001.

### 2.6 Behavioral analyses: accuracy

To evaluate if accuracy for the different trial types varied as a function of age cohorts (see Table 3), a 3 (group: younger, younger-old, older-old) × 5 (trial-type: Single, NS-comp, NS-incomp, SW-comp, SW-incomp) repeated measures ANOVA was conducted. There was a significant main effect of trial-type (*F*(4, 256) = 23.75, *MSE* = .16, *p* < .001, *η*_*p*_^*2*^ = .27). The pairwise comparisons, adjusted for Bonferroni corrections, found differences in accuracies for single task and all other dual task trial types (*p*′s < .02). However, accuracies for the two compatible trials types (NS-comp vs. SW-comp; *M*_*Difference*_ = 0.002, *SE* = 0.004, *p =* 1.00), and for the two incompatible trial types (NS-incomp vs. SW-incomp; *M*_*Difference*_ = 0.022, *SE* = 0.012, *p =* .622) were equivalent. The main effect of group was not significant (*F*(2, 64) = 2.60, *MSE* = .05, *p* = .08, *η*_*p*_^*2*^ = .08), but the group x trial-type interaction was significant (*F*(8, 256) = 2.81, *MSE* = .02, *p* = .045, *η*_*p*_^*2*^ = .08), although both the linear contrast (*F*(2, 64) = 3.18, *MSE* = .03, *p* = .048, *η*_*p*_^*2*^ = .09) and the cubic contrast (*F*(2, 64) = 4.64, *MSE* = .01, *p* = .01, *η*_*p*_^*2*^ = .13) were significant.

**Table 3:** Mean accuracy (SD) for the five trial types for each age group.

Trial-Type	Young *M (SE)*	Younger-Old *M (SE)*	Older-Old *M (SE)*
Single	0.97 (.01)	0.97 (.01)	0.96 (.04)
NS_Comp	1.00 (.01)	0.99 (.02)	0.99 (.03)
S_Comp	0.95 (.05)	0.86 (.20)	0.92 (.15)
NS_Incomp	0.99 (.02)	1.00 (.01)	0.99 (.02)
S_Incomp	0.95 (.08)	0.83 (.21)	0.89 (.16)

### 2.7 Brain-behavior relationships between accuracy, cognitive cost-sensitive ROIs, age-sensitive ROIs and whole brain correlation ROIs

Brain-behavior relationships, exhibited by the correlations between the ROIs and accuracy, were limited to only those costs that showed age-related differences in accuracy accuracies (Single and Dual trials for GSC, NS and SW trials for LSC). These relationships are shown for young, younger-old, and older-old separately to visualize any age-related differences in the patterns of brain-behavior relationships.

**Fig. 3:**
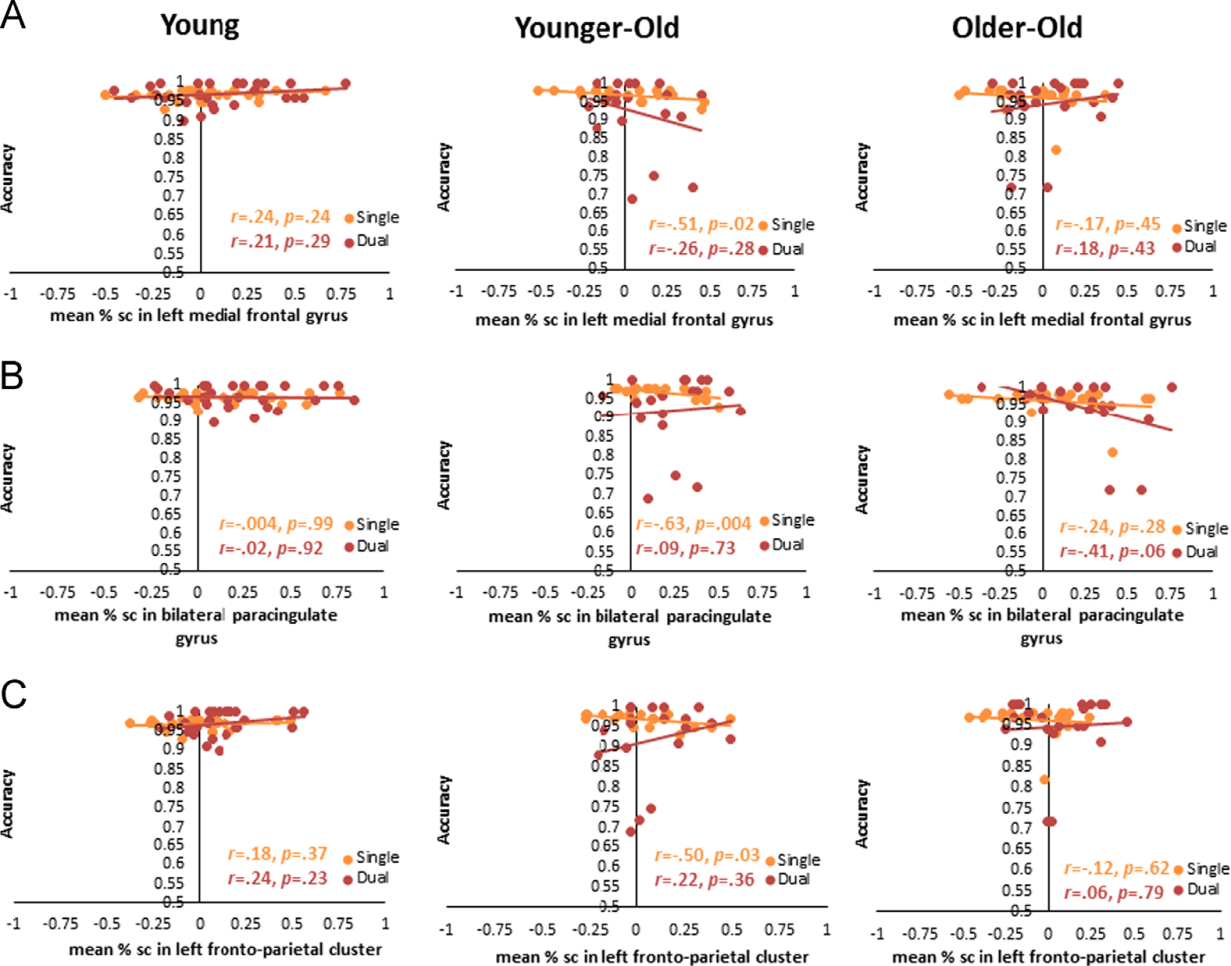
Brain-Behavior correlations between the percent signal change for the global switch cost-sensitive regions and accuracies in the corresponding blocks (i.e., Single and Dual). (A) Left medial frontal gyrus. (B) Bilateral paracingulate gyrus. (C) Left fronto-parietal cluster.

Three of the five GSC sensitive brain regions showed age-related related differences in the patterns of brain-behavior relationships for Single accuracy (Fig. 3). Higher Single accuracy was significantly related to less recruitment of GSC regions for Dual > Single contrast in younger-old (left medial frontal gyrus: *r* = − .51, *p* = .02; bilateral paracingulate gyrus: *r* = − .63, *p* < .01; left fronto-parietal cluster: *r* = − .50, *p* = .03).

**Fig. 4:**
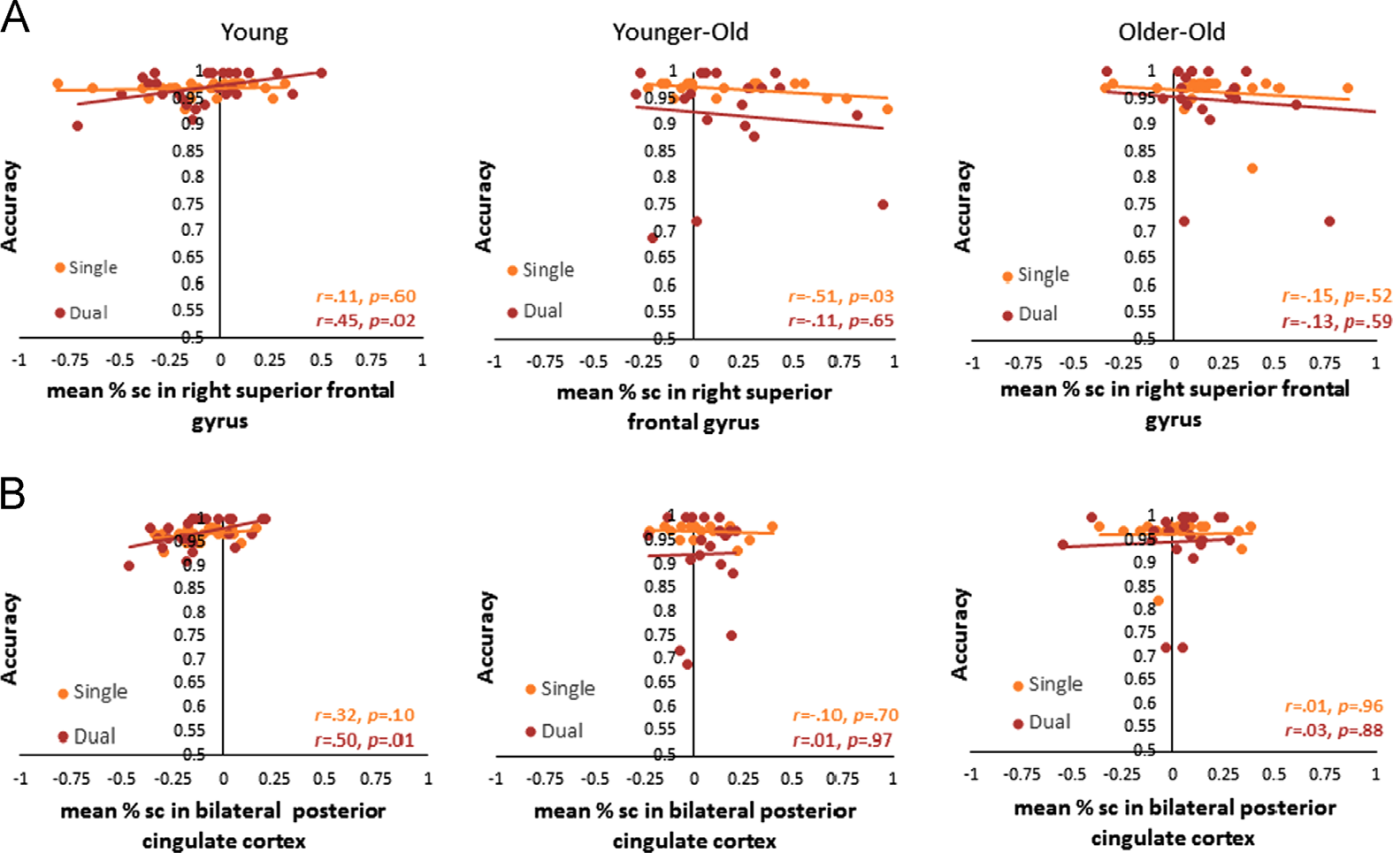
Brain-Behavior correlations between the percent signal change for the age-sensitive regions under-recruited in older adults and accuracies in the Single and Dual blocks. (A) Right superior frontal gyrus. (B) Bilateral paracingulate gyrus.

For the two regions that exhibited under-recruitment by older adults compared to younger adults, only one region (right superior frontal gyrus) showed a significant negative relationship between accuracy in single task and lower recruitment of the region for Task > Fixation contrast (Fig. 4) in younger-old.

**Fig. 5:**
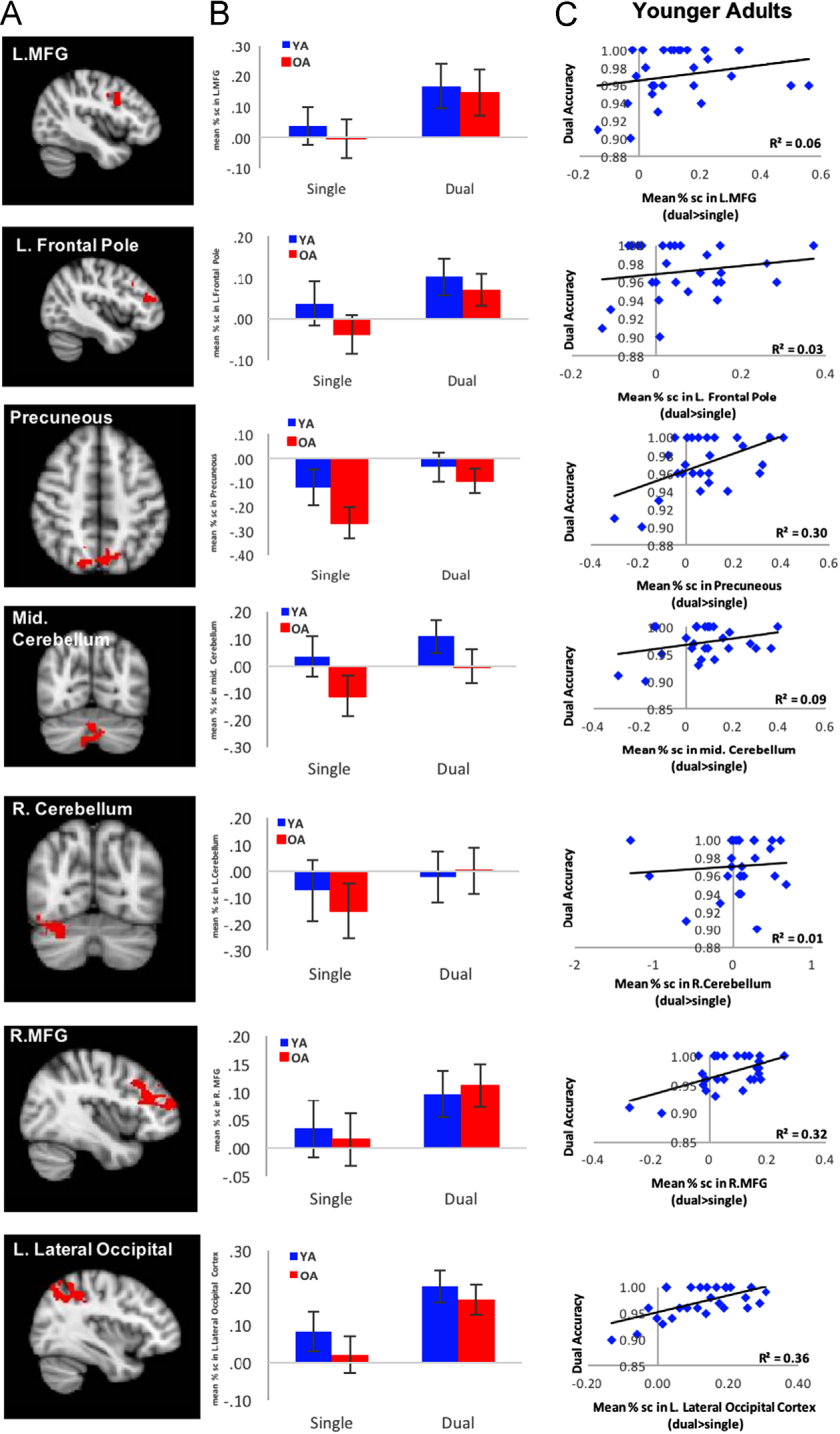
A) GSC sensitive brain regions that were significantly predicted by Dual accuracy in older adults. B) Age-group differences in these regions across single and dual task blocks. There were no significant effects of age-group or age-group × condition interactions for any of these 7 regions. The main effect of condition (Dual vs. Single) was significant for all regions. C) Correlations between activations of these GSC sensitive brain regions and Dual accuracy in younger adults only.

Out of the seven regions, which showed a positive relationship between GSC activation and Dual accuracy in older adults (Fig. 5), three regions showed significant correlations with Dual accuracy in younger adults: the bilateral precuneous (*r* = 0.55, *p* = .003), the right middle frontal gyrus (*r* = 0.57, *p* = .002), and the left lateral occipital cortex (*r* = 0.60, *p **=*** .001). Four brain regions showed exclusive compensatory recruitment patterns in older adults: left middle frontal gyrus, left frontal pole/middle frontal gyrus/inferior frontal gyrus, middle cerebellum and right cerebellum.
