# Supplementary material for: Functional magnetic neuroimaging data on age-related differences in task switching accuracy and reverse brain-behavior relationships
Source: Data Brief. 2018 May 18;19:997–1007. doi: 10.1016/j.dib.2018.05.059 (PMC5997903; doi:10.1016/j.dib.2018.05.059)
Supplement: Supplementary file 1 — Transparency document [file mmc1.docx]

No Conflict.
